# AMH Concentration and Gene Expression Level in Each Follicle and Their Relationship With Fertilization and Blastocyst Formation in Intracytoplasmic Sperm Injection

**DOI:** 10.1002/rmb2.12701

**Published:** 2025-11-26

**Authors:** Asuka Takeda, Yuri Yamamoto, Shota Yamamoto, Moeka Arata, Hina Eguchi, Noriko Hayashi, Yuya Yano, Hiroki Noguchi, Kou Tamura, Saki Minato, Hiroaki Inui, Tomohiro Kagawa, Riyo Kinouchi, Kanako Yoshida, Takeshi Iwasa

**Affiliations:** ^1^ Department of Obstetrics and Gynecology, Graduate School of Biomedical Sciences Tokushima University Tokushima Japan; ^2^ Department of Renal and Genitourinary Surgery, Graduate School of Medicine Hokkaido University Sapporo Japan

**Keywords:** anti‐mullerian hormone, clinical outcomes, follicular fluid, granulosa cells, intracytoplasmic sperm injection

## Abstract

**Purpose:**

The present study examined the relationship between Anti‐Mullerian hormone (AMH) concentrations in follicular fluid (FF) and AMH gene expression levels in granulosa cells (GCs) and the embryological outcomes of retrieved oocytes.

**Methods:**

Patients who underwent intracytoplasmic sperm injection (ICSI) treatment at our hospital were included. A total of 270 FF samples from 20 patients were used to evaluate AMH concentrations. A total of 140 GCs obtained during oocyte denudation for ICSI were used to assess AMH mRNA expression. The relationships between FF AMH concentrations or GC AMH mRNA expression levels and the maturity of the oocytes obtained, as well as fertilization and blastocyst arrival, were investigated.

**Results:**

FF AMH concentrations and GC AMH mRNA expression levels markedly varied among follicles. There was no correlation in the degree of variation in FF AMH concentration and GC AMH mRNA expression. Serum AMH concentrations correlated with mean FF AMH concentrations (*r* ^2^ = 0.78, *p* < 0.05), whereas no correlation was observed between follicular size and the FF AMH concentration. FF AMH concentrations were significantly lower in fertilized oocytes than in unfertilized oocytes, whereas GC AMH mRNA expression levels did not correlate with clinical outcomes.

**Conclusion:**

The FF AMH concentrations may be associated with oocyte fertilization capacity.

## 1 Introduction

Anti‐Mullerian hormone (AMH) is expressed in ovarian granulosa cells (GCs) in women and is an important regulator of follicular growth in mammals [1]. GCs' expression of AMH takes place in a specific window of folliculogenesis that starts shortly after the primordial follicle has embarked on development [2, 3]. AMH is generally expressed at low levels in primary follicles, increases to maximum concentrations in preantral and antral follicles, and then decreases with follicle development. The decrease in AMH as the cycle progresses releases follicles from its inhibitory effects on folliculogenesis and promotes maturation [4]. On the other hand, the expression of AMH is maintained at high levels in human cumulus cells even during the final stages of folliculogenesis [5]. Furthermore, AMH has been reported to be involved in steroid biosynthesis by the GCs, and elevated serum estradiol (E2) levels are associated with AMH receptor polymorphism in GCs [6]. This AMH form polymorphism has also been observed between follicles of the same size in the same woman [7], suggesting the existence of inter‐follicular variability in AMH concentration.

The AMH produced within the follicle appears to be partly accumulated in the follicular fluid (FF) and partly released into the bloodstream [2, 3]. Serum AMH is an important indicator of the ovarian reserve in reproductive‐age women [8]. In assisted reproductive technology (ART), serum AMH is used to counsel regarding the expected number of oocytes at retrieval, and also to select the ovarian stimulation protocol and required gonadotropin dosage. However, serum AMH does not correlate with pregnancy success, suggesting that it is a marker of the quantity, but not of the quality, of oocytes [8, 9, 10, 11].

FF AMH has been associated with ART clinical outcomes. Furthermore, FF AMH was shown to affect fertilization and pregnancy rates [12, 13, 14, 15, 16, 17, 18, 19, 20]. Similarly, some studies examined the relationship between the expression of AMH in GCs and ART clinical outcomes [21, 22, 23]. However, most of these studies were based on measurements from 1 to 3 follicles per individual or pooled FF, and the study designs and findings obtained were inconsistent. Limited information is currently available on the degree of variation per follicle within an individual and its relationship with the clinical outcomes of individual fertilized oocytes. Schenk et al. [24] examined FF collected from individual follicles within one stimulated cycle and found that FF AMH differed between patients; however, individual FF AMH values correlated with serum AMH concentrations in the respective patients. Therefore, the present study investigated variations in FF AMH concentrations and AMH gene expression levels among developing follicles. In addition, the relationships between these factors and the clinical outcomes of retrieved oocytes were examined.

## 2 Methods

### 2.1 Study Design

Infertile patients who were scheduled for intracytoplasmic sperm injection (ICSI) treatment between March 2023 and November 2024 were enrolled. Patients with serum AMH < 0.5 ng/mL, polycystic ovarian syndrome, and aged 43 years and older were excluded from the study. Written informed consent was obtained from all subjects after they had received an explanation about the study. This prospective cohort study was approved by the clinical research review board of Tokushima University (Approved No. 4315). The aim of the present study was to evaluate variations in FF AMH among individuals and the relationships between FF AMH and related oocyte embryological outcomes. GC AMH mRNA expression levels were similarly examined in relation to embryological outcomes.

### 2.2 Ovarian Stimulation Protocols

Patients were scheduled for controlled ovarian hyperstimulation, involving either a gonadotrophin‐releasing hormone (GnRH) agonist‐ or antagonist‐based protocol, or Progestin‐primed Ovarian Stimulation (PPOS). In the GnRH agonist‐based protocol, patients were started on buserelin acetate in the mid‐luteal phase of the preceding cycle. The administration of follicle‐stimulating hormone (FSH) or human menopausal gonadotrophin was initiated, and the initial dose was adjusted on the basis of the patient's antral follicular count, age, and serum AMH level and was maintained for approximately 5 days. Subsequent gonadotropin doses were modified to the ovarian response. In the GnRH antagonist‐based protocol, ganirelix treatment was started when the dominant follicle reached > 14 mm in diameter. In the PPOS protocol, progesterone was administered orally at the same time as the initiation of the FSH treatment. When the follicle size reached approximately 18 mm, recombinant human chorionic gonadotrophin (hCG) 250 μg was administered. Approximately 36 h after the administration of hCG, transvaginal ultrasound‐guided oocyte pick‐up (OPU) was performed. Prior to aspiration, follicle diameter was measured in one direction using ultrasound (KONICA MINOLTA Inc., SONOVISTA GX30, Japan). FF was manually aspirated, and the syringe volume was recorded as the follicular fluid volume. Oocytes were related to their unique follicle hormone concentration. The retrieved oocytes were visually assessed for maturity as either MeiosisII (MII) oocytes or non‐MII oocytes (Germinal vesicle [GV] stage, MetaphaseI [MI] stage, or atretic), and MII oocytes were identified by the presence of the first polar body. In all cases, ICSI was performed because of a male factor or a previous history of improved fertilization rates with ICSI. Fertilized oocytes were cultured individually at the blastocyst stage (day 5 or 6).

### 2.3 Serum and FF Collection

Serum samples were obtained before ovarian hyperstimulation. FF was collected from all follicles punctured during each patient's oocyte retrieval to minimize contamination with blood during the procedure. Unless there was a specific reason, the puncture needle was not replaced. Before puncturing, the follicle diameter was measured in one direction by ultrasound and the FF volume aspirated was recorded. FF samples were centrifuged at 2000 rpm at room temperature for 5 min, and the supernatant was collected and stored at –80°C until assayed. The measurement of FF AMH level was performed independently for each FF sample. FF AMH levels were measured by FUJIREBIO Inc. (Hachioji City, Tokyo, Japan) under the joint research agreement, and the limit of detection for AMH was 0.024 ng/mL. Serum and FF AMH levels were measured using enzyme‐linked immunosorbent assays (FUJIREBIO Inc., LUMIPULSE, Japan).

### 2.4 Granulosa Cell Extraction, RNA Extraction, and Real‐Time Polymerase Chain Reaction (PCR)

GCs were obtained during oocyte denudation for ICSI. After oocyte retrieval, GCs were removed from the cumulus–oocyte–complex with hyaluronidase. GCs were not recovered from the follicular fluid. Cells were centrifuged at 2000× *g* at room temperature for 5 min, and the resulting pellets were subjected to RNA extraction. Total mRNA was isolated using a TRIzol reagent kit (Invitrogen Co., Carlsbad, CA, USA) and RNeasy mini kit (Qiagen GmbH, Hilden, Germany). cDNA was synthesized with oligo (deoxythymidine) primers at 50°C using the SuperScript III First‐Strand Synthesis System for Real‐Time PCR (Invitrogen Co., Life Technologies Japan Ltd., Minato Ward, Tokyo, Japan), Real‐Time PCR System (PE Applied Biosystems, Foster City, CA, USA), and Fast SYBR Green (Invitrogen Co.).

AMH mRNA concentrations were calculated relative to the β‐actin mRNA concentration in the same sample. A melting curve analysis was also performed for each gene at the end of PCR. Each amplicon generated a single peak. Primer sequences and annealing temperatures are shown in Table 1. PCR conditions were as follows: initial denaturation and enzyme activation at 95°C for 20 s, followed by 45 cycles of denaturation at 95°C for 3 s, and annealing and extension for 30 s. AMH gene expression was analyzed for each oocyte, and cumulative values were not used. AMH mRNA expression in GCs was converted to median values per patient to show intra‐individual variability.

**TABLE 1:** Primer sequences and annealing temperatures.

Primer	Sequence	Annealing T (°C)
AMH forward	CCT TCC ACT CGG CTC ACT TA	60°C
AMH reverse	AGG CCA GTC CAA GTC TTC TC	
β‐Actin forward	CCT GGA CTT CGA GCA AGA GA	60°C
β‐Actin reverse	CAG CGG AAC CGC TCA TTG CCA ATG G	

### 2.5 Statistical Analysis

All results are expressed as the mean ± standard error of the mean. Data analyses were performed using a one‐way ANOVA followed by the Student's *t*‐test. *p*‐values < 0.05 were considered to be significant.

## 3 Results

Patients' clinical backgrounds are summarized in Table 2. Twenty patients (age 37.0 ± 0.9 years) were enrolled in this study. The mean number of oocytes retrieved was 10.3 ± 1.2, varying from patient to patient. The mean serum AMH concentration was 3.4 ± 0.5 ng/mL, and the mean FF AMH concentration was 1.5 ± 0.3 ng/mL, which was lower than the mean serum AMH concentration. The mean FF AMH according to treatment protocol was 1.4 ± 0.7 ng/mL for GnRH agonists, 1.6 ± 0.3 ng/mL for GnRH antagonists, and 2.0 ± 1.1 ng/mL for PPOS, with no significant differences observed.

**TABLE 2:** Patient characteristics.

age [year]	37.0 ± 0.9
Gravida	1.1 ± 0.4
Para	0.3 ± 0.1
BMI	22.5 ± 1.0
Cause of infertility	
Male, *n* (%)	12 (60)
Tubal, *n* (%)	2 (10)
Unexplained, *n* (%)	6 (30)
Treatment protocol	
Agonist, *n* (%)	3 (15)
Antagonist, *n* (%)	16 (80)
PPOS, *n* (%)	1 (5)
Serum AMH level [ng/ml]	3.4 ± 0.5
TVS diameter of aspirated follicle [mm]	19.9 ± 0.3
Volume of aspirated FF [mL]	3.9 ± 0.1
Number of oocyte retrived	10.3 ± 1.2
Embryological outcomes	
Mature oocyte rate (%)	67.9 ± 4.0
Fertilization rate (%)	74.1 ± 5.0
Blastocyst arrival rate (%)	38.7 ± 5.6
Clinical outcomes	
Pregnancy per ET, *n* (%)	7/20 (30.4)

Abbreviations: AMH, anti‐mullerian hormone; BMI, body mass indes; ET, embryo transfer; FF, follicular fulid; PPOS, progestin‐primed ovarian stimulation; TVS, transvaginal ultrasounds.

A total of 270 follicles were obtained, and 160 oocytes were collected, of which 108 (67.5%) were MII oocytes, 80 (74.1%) were fertilized, and 37 (46.3%) developed into blastocysts. GCs were obtained from 140 oocytes. By follicle size, there were 13 follicles (4.8%) measuring 13 mm or less, 146 follicles (54.1%) measuring 14–20 mm, and 111 follicles (41.1%) measuring 21 mm or more. The smallest follicle diameter after puncture was 9 mm.

**FIGURE 1:**
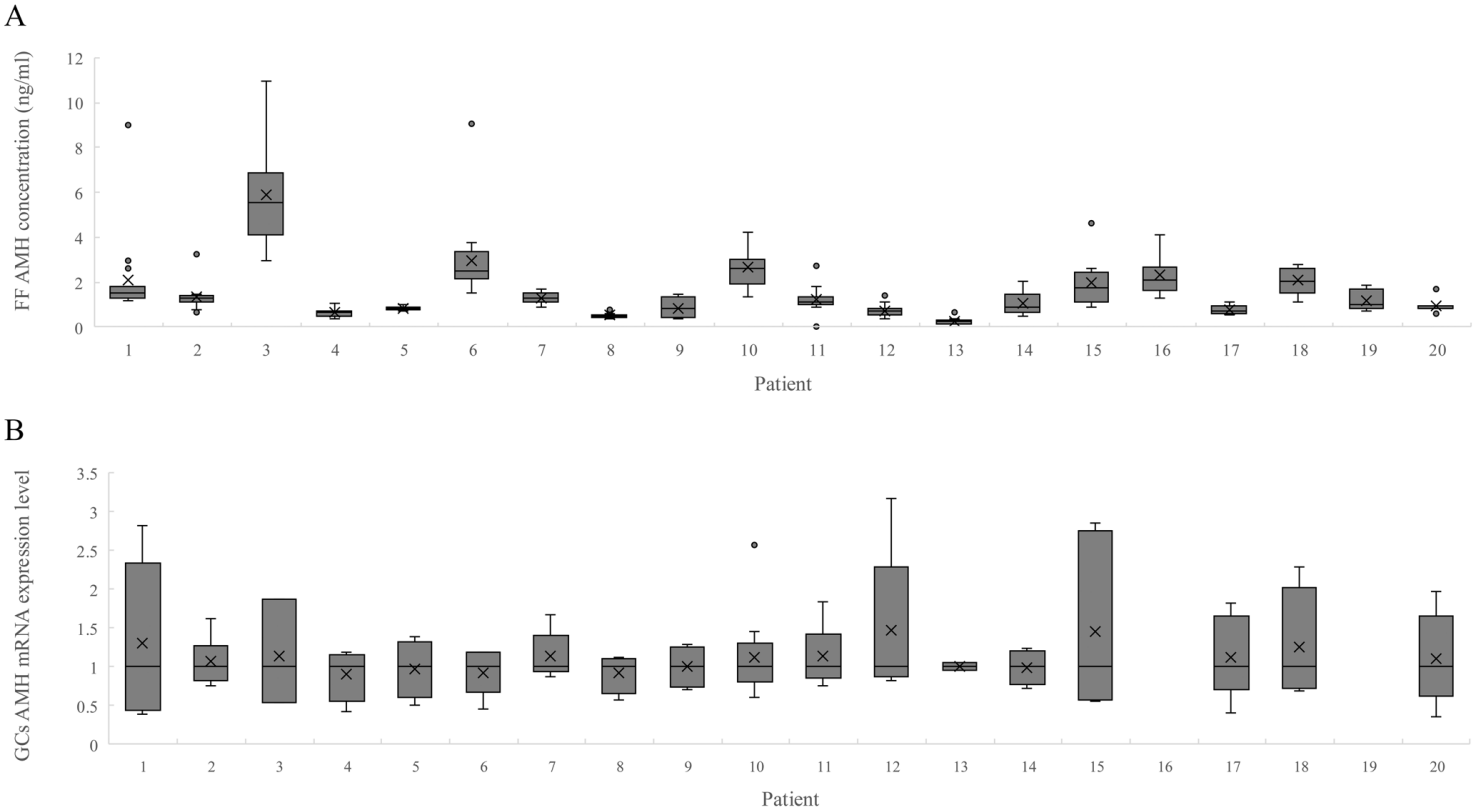
AMH concentration in the follicular fluid of each patient. (A) FF AMH concentrations and (B) GC AMH mRNA expression levels in each patient. The median is expressed as bars in the graph, and the mean as ×. AMH mRNA concentrations were calculated relative to the β‐actin mRNA concentration in the same sample. Mean values for each patient are expressed as 1.0. ND, not detected.

Variations were observed in FF AMH concentrations among growing follicles within each patient, and the degree of variation differed between individuals. Variations in FF AMH within individuals were larger in patients with a high mean FF AMH (Figure 1A). GC AMH mRNA expression levels varied from follicle to follicle; however, the degree of variation differed among each patient and from FF AMH variations (Figure 1B). There was no correlation found between FF AMH concentrations and GC AMH mRNA expression levels.

**FIGURE 2:**
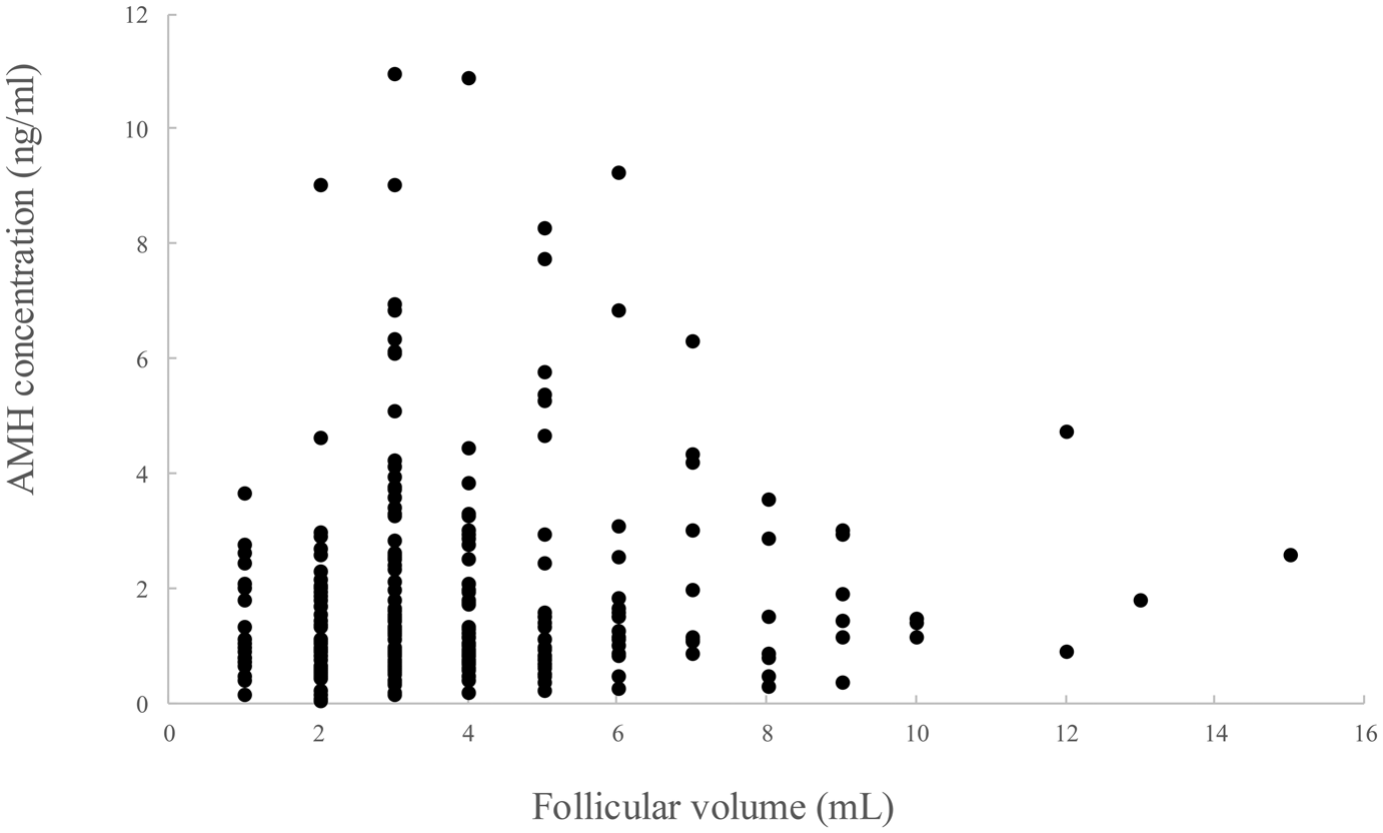
Relationship between the FF AMH concentration and follicular size. Relationship between the FF AMH concentration and (A) follicular diameter or (B) FF volume. FF, follicular fluid.

FF AMH concentration was not correlated with follicular diameter nor follicular fluid volume (Figure 2A,B). Similarly, no correlation was observed between GC AMH mRNA expression and follicle diameter or follicular fluid volume. The cutoff value for follicular volume was 1 mL. FF AMH and GC AMH were each divided into two groups on the basis of follicular volume and analyzed. FF AMH was 1.8 ± 0.2 ng/mL in the group with follicle volume of 3 mL or less (149 follicles) and 1.9 ± 0.2 ng/mL in the group with follicle volume of 4 mL or more (121 follicles). GC AMH mRNA expression levels were 1.8 ± 0.3 in the group with follicular volume of 3 mL or less (68 samples) and 1.1 ± 0.1 in the group with follicular volume of 4 mL or more (53 samples); no significant differences were observed between the two groups. There were no differences in FF AMH concentration or GC AMH mRNA expression depending on follicular volume.

**FIGURE 3:**
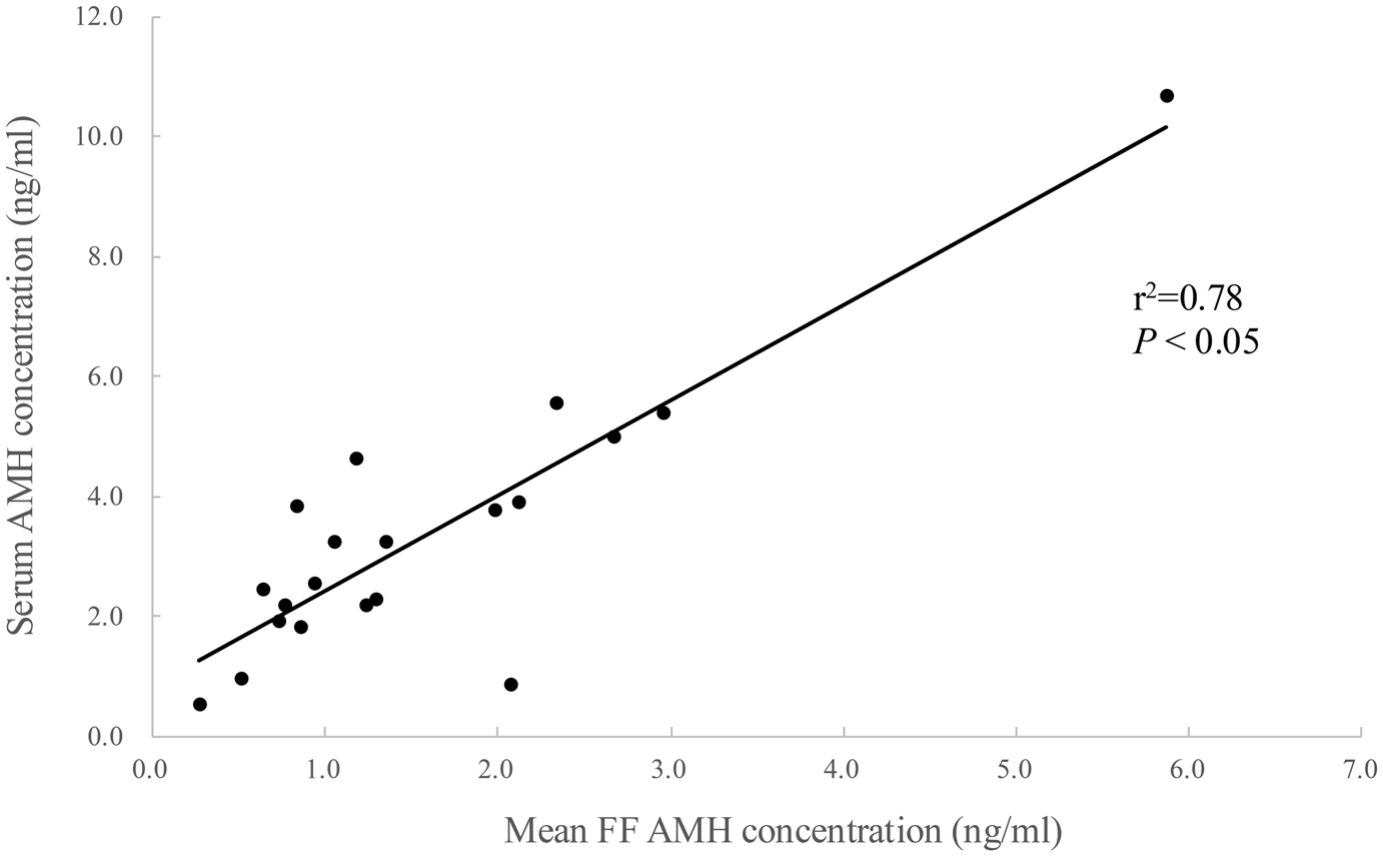
Relationship between mean FF and serum AMH concentrations. FF, follicular fluid.

A significant positive correlation was observed between serum AMH concentration and mean FF AMH concentration (*r* ^2^ = 0.78, *p* < 0.05) (Figure 3).

**FIGURE 4:**
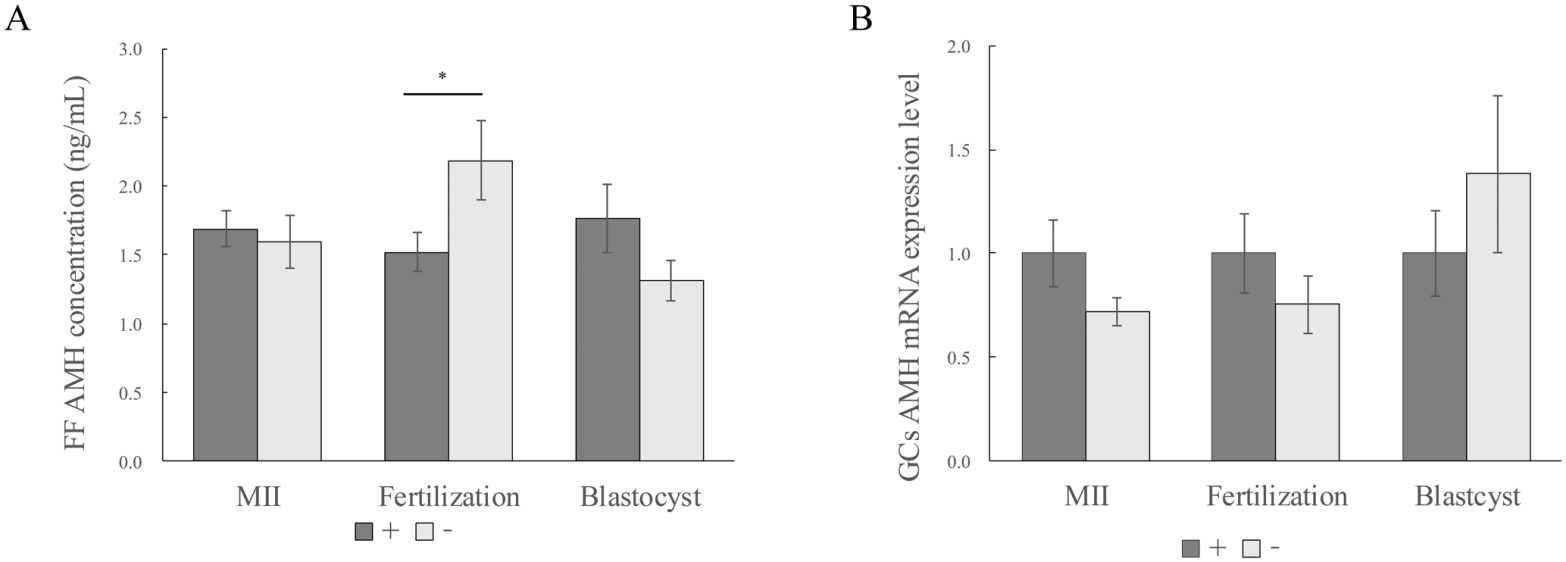
Relationship between FF or GC AMH mRNA expression and clinical outcomes. Comparison of (A) FF AMH concentrations and (B) GC AMH mRNA expression levels corresponding to oocytes for each embryological outcome (MII oocyte, fertilization, and blastocyst arrival). AMH mRNA concentrations were calculated relative to the β‐actin mRNA concentration in the same sample. Mean values of the + group are expressed as 1.0. FF, follicular fluid; GCs, cumulus granulosa cells. *p < 0.05.

AMH concentrations in FF collected from follicles with fertilized oocytes were significantly lower than from those with unfertilized oocytes (1.5 ± 0.1 vs. 2.2 ± 0.3, *p* = 0.04), whereas no significant difference was observed in AMH concentrations in FF collected from follicles with mature and immature oocytes (Figure 4A). Similarly, no significant difference was noted in AMH concentrations in FF collected from follicles with oocytes that developed into blastocysts and non‐developed oocytes (Figure 4A). On the other hand, AMH mRNA expression levels in GCs did not significantly differ regardless of embryological outcomes (Figure 4B).

FF AMH concentrations were shown according to oocyte maturity (Table 3), but there was no significant difference in FF AMH between fertilized oocytes and immature oocytes (GV or MI). The relationship between follicle size and oocyte maturity was also examined, but no differences were observed in either follicle diameter or follicular volume.

**TABLE 3:** Follicular fluid AMH according to oocyte maturity.

Maturity stage of oocytes (*n*)	FF AMH [ng/mL]
GV (8)	2.31 ± 0.39
MI (17)	2.29 ± 0.55
Atretic (28)	1.23 ± 0.16
MII (104)	1.62 ± 0.12
Fertirization (−) (26)	2.19 ± 0.29
Fertirization (+) (78)	1.51 ± 0.14
Blastcyst (−) (42)	1.31 ± 0.14
Blastcyst (+) (36)	1.76 ± 0.25

*Note:* There was no significant difference in follicular fluid AMH between MII oocytes and immature oocytes (GV or MI).

Abbreviations: FF, follicular fluid; GV, germinal vesicle; MI, metaphase I; MII, meiosis II.

## 4 Discussion

In the present study, we measured FF AMH concentrations and GC AMH mRNA expression levels in each follicle and examined their relationship with follicular sizes and the embryological outcomes of the corresponding oocytes. The results obtained revealed variations in FF and GC AMH levels among developing follicles within an individual, and mean FF AMH concentrations correlated with serum AMH concentrations. Furthermore, FF AMH concentrations in FF collected from follicles with fertilized oocytes were significantly lower than those with unfertilized oocytes, whereas GC AMH mRNA expression levels did not significantly differ.

Schenk et al. [24] demonstrated that mean FF AMH concentrations correlated with serum AMH concentrations and did not significantly differ among patients. Similarly, mean FF AMH concentrations correlated with serum AMH concentrations and variations were observed in FF AMH concentrations among developing follicles within an individual. Therefore, serum AMH concentrations appear to reflect FF AMH concentrations and cases of high FF AMH concentrations showed slightly more variation. Sacha et al. and Schenk et al. [14, 24] reported that FF AMH concentrations were higher than serum AMH concentrations; however, the present results revealed that FF AMH concentrations were lower than serum AMH concentrations. Although serum AMH concentrations are known to be stable [8, 25], Mamsen et al. [7] detected several AMH forms in FF and GCs, indicating that it is difficult to standardize AMH because of its complex and variable intracellular processing in follicles. FF AMH concentrations may lead to more inter‐follicular variations and be less stable than serum AMH concentrations, which may be the reason for differences in the findings of each study. Although previous studies only used FF from the first punctured follicle per ovary or patient, it may be difficult to investigate the roles of FF AMH from a small number of follicles because of the high variability in FF AMH concentrations and gene expression. Further investigations are needed on the collection and storage of FF and the detection of FF AMH.

Intrafollicular concentrations of AMH become progressively lower as follicle diameters increase; however, in this study, FF AMH concentrations did not correlate with FF volume. Andersen et al. examined follicles measuring 3–12 mm and demonstrated FF AMH concentrations decreased when follicles reached ≥ 9 mm, with AMH being an important intrafollicular mediator of follicular development before the follicular selection phase [4]. Weenen et al. [26] demonstrated a higher level of AMH expression was present in the GCs of secondary, preantral and small antral follicles, which supports this finding. On the other hand, Poulsen et al. [27] investigated fluctuations in hormone levels in FF during the ovulation period and showed that the concentration of AMH in FF decreased after the administration of ovulation‐inducing drugs, suggesting that the role of AMH in FF is negligible. In small follicles prior to follicle selection, AMH functions as a regulatory factor for follicle development, and AMH concentrations in FF are higher. However, in mature follicles following the administration of ovulation‐induction drugs, AMH concentrations in FF may be lower. In the present study, most of the follicles punctured for OPU were mature dominant follicles measuring ≥ 14 mm after the induction of ovulation, and we speculate that FF AMH may not be affected by FF size among mature follicles after oocyte maturation.

In the present study, multiple oocyte FF pairs were collected from one patient. The embryological outcomes were compared at each stage of the mature oocyte retrieval, fertilization and blastocyst arrival, and FF AMH concentrations were significantly lower in fertilized oocytes than in unfertilized oocytes. Previous studies examined the relationship between FF AMH concentrations and laboratory outcomes; however, the findings obtained were controversial. Milaković et al. [18] collected FF from a single dominant follicle in each patient and showed that FF AMH concentrations were significantly lower in fertilized MII oocytes than in non‐fertilized MII oocytes in patients younger than 35 years. These findings are consistent with the present results. On the other hand, Kim et al. [12] examined FF collected from the largest preovulatory follicle of each ovary, divided samples into three groups on the basis of FF AMH concentrations, and showed that the fertilization rate was significantly lower in the low group than in the intermediate group. Lv et al. [19] demonstrated that FF AMH concentrations did not affect outcomes. The findings of these studies cannot be compared because of differences in FF conditions, and it is difficult to relate clinical results to FF from a few dominant follicles per individual or pooled FF. Ciepiela et al. [20] examined clinical outcomes using oocyte‐matched FF from the first puncture and showed that FF AMH concentrations were higher in follicles in which oocytes developed blastocysts and led to live births than in follicles that were unfertilized; therefore, FF AMH was identified as a predictor of live births.

Research on fertilization failure is extensive, and although the impact of oocyte maturation processes [28, 29] and the cumulus cells [30] is known, it cannot be explained by a single factor. The role of AMH remains unclear, but it may serve as a marker for GCs' function. Previous studies showed that AMH has a suppressive role in the regulation of follicular development [31, 32], and the presence of oocytes affects AMH expression in GCs [33]. Dickman et al. [21] demonstrated that GC AMH mRNA expression levels and FF AMH concentrations were significantly higher in GV and MI oocytes than in MII oocytes, suggesting AMH involvement in meiosis. Although no significant difference was observed in this study, a similar trend was noted for FF AMH concentration. Furthermore, AMH and E2 concentrations inversely correlated in FF [17], and a high FF E2 concentration increased the chances of pregnancy [34]. Oocyte fates are strongly dependent on the corresponding follicles, particularly GCs [35], and follicles with lower FF AMH concentrations are more likely to have more advanced follicle development and higher E2 concentrations, leading to more advanced LH surge effects and oocyte maturation.

On the other hand, FF AMH did not show any difference in relation to blastocyst arrival. O'Brien et al. [15] demonstrated that AMH and progesterone (P4) in FF are associated with blastocyst arrival. Additionally, several studies have suggested that FF P4 is involved in implantation [36, 37]. Other factors besides AMH in FF may also significantly influence post‐fertilization outcomes. In the present study, there were no differences in the expression of AMH in GCs on the basis of the embryological outcome. Previous studies examined factors that affect FF AMH concentrations [23, 38, 39, 40, 41]. Wolff et al. [23] investigated the effects of stimulation methods and showed a correlation between FF and GC AMH concentrations in non‐stimulated cycles, but not in stimulated cycles. This suggests that disruption of the follicular fluid hormone environment caused by ovarian stimulation may lead to abnormal AMH production [23, 42]. Furthermore, this may also be influenced by the fact that AMH synthesis is highly complex and difficult to identify [7]. A previous study using ovarian sections has demonstrated higher AMH expression in granulosa cells within the cumulus‐oophorous complex than those in other regions [26]. Therefore, GCs obtained from isolated cumulus cells may not accurately reflect follicular AMH mRNA expression. FF AMH concentration may be superior to GC AMH mRNA expression levels for evaluating AMH within follicles. Particularly in controlled ovarian stimulation, FF AMH concentrations may better reflect oocyte outcomes than GC AMH mRNA expression levels.

There are a number of limitations that need to be addressed. The sample size was small. Furthermore, since the present study was limited to cases treated with ICSI, the male factor may have had an impact on laboratory outcomes. In addition, biases may have arisen because of differences in infertility causes and treatment protocols, not just male factors. Furthermore, there were many frozen embryos, and not all of them could be transferred, so the sample size was too small to evaluate pregnancy rates, miscarriage rates, and birth rates. Long‐term observations after embryo transfer are needed to clarify the relationship between pregnancy and perinatal outcomes. In some patients, there were few good‐quality oocytes, and in some cases, blastocysts could not be obtained; therefore, it was difficult to analyze clinical outcomes on the basis of oocytes from the same patient. Since FF was collected in accordance with the clinical OPU, there is a possibility that follicular fluid remained in the puncture needle or that culture medium was mixed in. In addition, measuring hormone levels other than AMH in individual follicular fluid samples may have provided more valuable data for evaluating each follicle individually.

Few studies have individually cultured oocytes and investigated the relationship between FF AMH concentrations and laboratory outcomes. The fact that FF was collected individually from all punctured follicles during OPU and analyzed may have an important impact on future FF research. The evaluation of serum AMH concentrations is a common test and is useful for predicting responses to a stimulation in ART [8, 43]. In this study, differences in FF AMH concentrations were found to correlate with the fertilization of corresponding oocytes, suggesting that FF AMH may be a marker of oocyte quality. However, FF AMH concentrations and GC AMH mRNA expression levels are not as stable as serum AMH concentrations and, thus, require more effort to collect. In addition, long‐term follow‐up to examine pregnancy and perinatal outcomes would clarify the role of FF AMH. Therefore, further research is needed before FF and GC AMH concentrations can be clinically applied to ART.

In summary, we herein demonstrated variations in FF AMH concentrations within individuals and between follicles. FF AMH concentrations did not correlate with follicular volume, but correlated with serum AMH concentrations. Furthermore, FF AMH concentrations were lower in fertilized oocytes than in unfertilized oocytes and, thus, may affect the fertilization potential of oocytes.

## Ethics Statement

This study was approved by the clinical research review board of Tokushima University (Approved No. 4315). Dr. Takeshi Iwasa is an Editorial Board member of Reproductive Medicine and Biology and a co‐author of this article. To minimize bias, they were excluded from all editorial decision‐making related to the acceptance of this article for publication.

## Conflicts of Interest

The authors declare no conflicts of interest.

## Data Availability

The data that support the findings of this study are available on request from the corresponding author. The data are not publicly available due to privacy or ethical restrictions.
